# Unrecognized tibial nerve injury in total-ankle arthroplasty: Two case reports

**DOI:** 10.1097/MD.0000000000021474

**Published:** 2020-07-31

**Authors:** Gun-Woo Lee, Keun-Bae Lee

**Affiliations:** Department of Orthopedic Surgery, Chonnam National University Medical School and Hospital, Gwangju, Republic of Korea.

**Keywords:** complication, tibial nerve injury, total-ankle arthroplasty

## Abstract

**Rationale:**

Tibial nerve injury is a sustainable but rare complication during total-ankle arthroplasty (TAA). We outlined 2 previously unreported cases of tibial nerve injury in TAA, including the prognoses and possible causes.

**Patient concerns:**

First, a 63-year-old woman complained of a 5-month history of persistent tingling sensation and numbness on the medial and plantar aspects of her foot after TAA. Second, a 50-year-old woman complained of a 6-month history of tingling sensation and numbness on the plantar surface of her forefoot after TAA.

**Diagnosis:**

Explorations were performed on suspicion of tarsal tunnel syndrome; however, both patients exhibited complete laceration of tibial nerve with neuroma formation.

**Interventions:**

In both patients, we excised the neuroma and performed end-to-end nerve repair.

**Outcomes:**

The sensory disturbance of the sole considerably improved at long-term follow-up over 8 years after the neurorrhaphy procedures.

**Lessons:**

Tibial nerve injury is rare following TAA, and is sometimes unrecognized or misdiagnosed. If tibial nerve injury is suspected, prompt surgical exploration should be performed; great precaution must also be taken to prevent injury of the tibial nerve during TAA.

## 1 Introduction

Modern total-ankle arthroplasty (TAA) has shown satisfactory outcomes in patients with end-stage ankle arthritis.^[1]^ Some studies have reported on the difficulty of performing TAA, the steep learning curve, or various postoperative complications.^[2]^ Among the complications, tibial nerve injury rarely occurs in TAA.^[3]^ However, these injuries can produce serious problems ranging from pain and numbness to atrophy of the intrinsic plantar muscle.^[4]^ Few studies have reported on iatrogenic injury of the tibial nerve, and the possible etiologies of injury are rarely identified.^[5]^ Here, we report 2 cases of tibial nerve injury during TAA, including the prognoses, possible causes of injury, and a literature review.

## 2 Case description

This case report was approved by the institutional review board of our hospital, and the informed consent for publication was obtained from both patients.

### 2.1 Case 1

**Figure 1:**
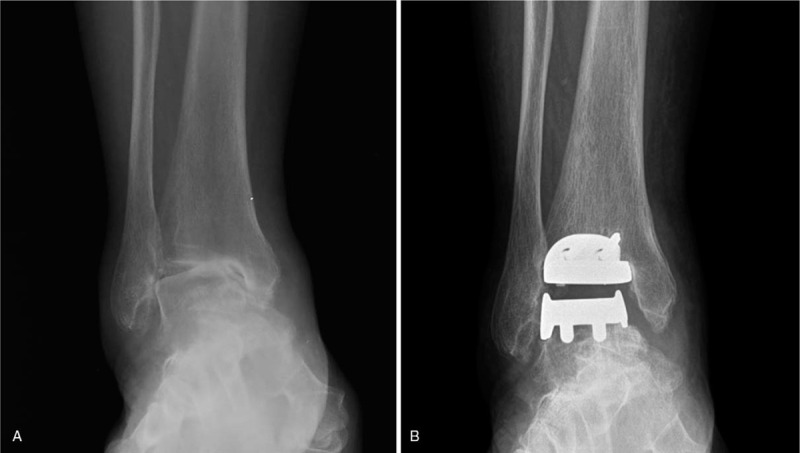
Weight-bearing anteroposterior radiographs of a 63-year-old woman. Preoperative radiograph indicating 21° of ankle incongruent varus deformity (A). Postoperative radiograph (B).

A 63-year-old woman presented with an 8-year history of ankle pain and prolonged lateral ankle instability. A plain radiograph showed a 21° ankle-incongruent varus deformity. We performed TAA with deltoid ligament release and a modified Broström procedure for end-stage osteoarthritis due to repeated ankle sprains (Fig. 1).

One month after the TAA, she complained of a persistent tingling sensation and numbness on the medial and plantar aspects of her foot. On physical examination, Tinel sign was positive for the tibial nerve behind the medial malleolus. The symptoms were refractory to conservative treatment, including a 4-month course of nonsteroidal anti-inflammatory drugs and gabapentin.

**Figure 2:**
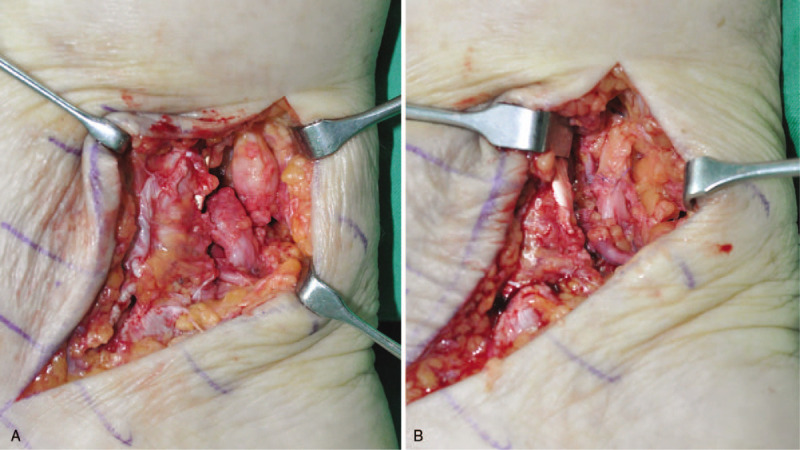
Intraoperative finding of tibial nerve injury. Complete laceration with neuroma formation (A). End-to-end nerve repair (B).

Five months after the TAA, we suspected tarsal tunnel syndrome and attempted to explore the posterior aspect of medial malleolus. During the exploration, we found complete laceration of the tibial nerve at the posteromedial corner of the ankle joint, with a neuroma formation at the injured stump. We excised the neuroma and performed end-to-end nerve repair (Fig. 2). Three months after nerve repair, her symptoms were partially improved but she continued to complain of tingling sensation and numbness on the plantar surface of the forefoot. For more than 8 years after neurorrhaphy, numbness on the plantar side of forefoot persisted, however, tingling sensation considerably improved.

### 2.2 Case 2

**Figure 3:**
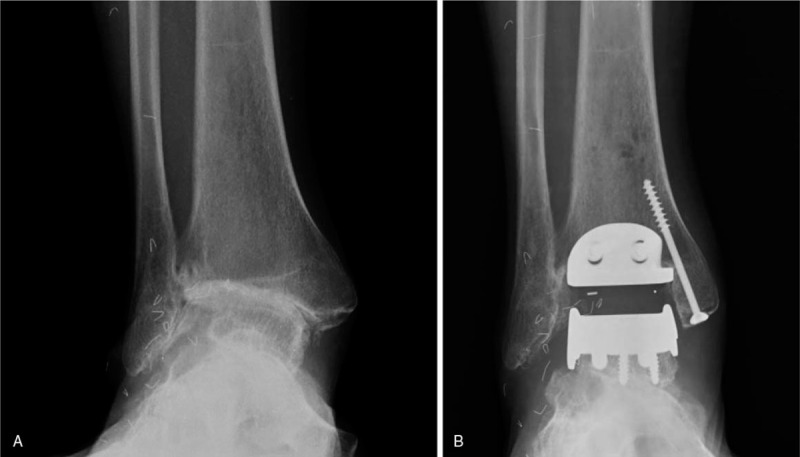
Weight-bearing anteroposterior radiographs of a 50-year-old woman. Preoperative radiograph indicating 14° of ankle-incongruent valgus deformity due to growth arrest in the lateral aspect of the distal tibia (A). Postoperative radiograph (B).

A 50-year-old woman presented with a 7-year history of ankle pain. The patient had a remote history of injury to the ankle involving physis of the distal tibia as a child. A plain radiograph showed a 14° ankle-incongruent valgus deformity due to growth arrest in the lateral aspect of the distal tibia. We performed TAA for posttraumatic osteoarthritis (Fig. 3).

**Figure 4:**
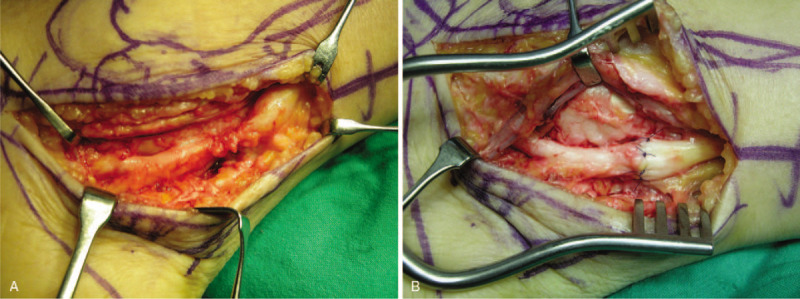
Intraoperative finding of tibial nerve injury. Complete laceration with neuroma formation (A). End-to-end nerve repair (B).

For 6 months after surgery, she complained of tingling sensation and numbness on the plantar surface of the forefoot. An electromyographic study indicated incomplete tibial neuropathy. We suspected tarsal tunnel syndrome and performed exploratory surgery. Similar to the previous case, we found complete laceration of the tibial nerve with neuroma formation. We excised the neuroma and performed a neurorrhaphy (Fig. 4). Sensory disturbance of the sole partially improved over 3 years after nerve repair. However, over than 11 years after neurorrhaphy, both tingling sensation and numbness of the sole considerably improved.

## 3 Discussion

Intraoperative tibial nerve injury may occur during TAA, but is a rarely reported complication. Tibial nerve injury can lead to serious problems ranging from pain and sensory disturbances to intrinsic muscle atrophy.^[6,7]^ To our best knowledge, no studies have investigated the possibility of intraoperative tibial nerve injury in TAA and its treatment and outcomes. Hence, we report 2 cases of tibial nerve injury with clinical outcomes. Additionally, we have tried to describe their possible causes.

Various circumstances may contribute to this complication. Anatomic alterations around the ankle joint may increase the possibility of iatrogenic injuries.^[7]^ Our first patient had a 21° ankle-incongruent varus deformity and suffered from instability for many years before TAA. This preoperative deformity may have altered the anatomy and pathway of the tibial nerve. The 2nd patient also had a 14° valgus deformity with growth arrest in the lateral tibial plafond. In addition to anatomic alterations, posttraumatic conditions surrounding the ankle may have rendered the tibial nerve more prone to intraoperative injury. Previous traumatic injuries can cause stenosis and scarring. Local scarring in the soft tissue can tether the nerve, making it less able to tolerate even minor changes. This is more of a problem in cases where posterior sclerosis and fibrosis are present.^[2,8]^

When visualizing the anatomy of the posterior ankle, we were able to speculate as to why the tibial nerve could have been harmed during surgery. This nerve is located behind the medial malleolus and is, therefore, near the tip of the saw blade during the tibial bone cut. More precisely, it is located 2 to 6 mm from the posterior surface of the tibia, making it prone to injury during surgery. At the level of the talus, the tibial nerve and artery were 5 to 11 mm from the posterior body of the talus.^[9,10]^ Therefore, surgery without consideration of this vulnerability may lead to iatrogenic injury of posterior ankle structures during TAA. In particular, tibial nerve injury can occur as a result of nerve contact with the saw blade while cutting the tibial bone at the posteromedial corner.^[4]^

The low reporting rate of tibial nerve injury may be attributable to several factors. These nerve injuries tend to go unrecognized during and after surgery. Even if nerve injury is suspected, diagnosis may be delayed because tibial nerve injury is often misdiagnosed as tarsal tunnel syndrome due to the similarity in symptoms. Thus, rather than immediate surgical treatment, conservative treatment is usually performed first.

To avoid this serious iatrogenic injury during TAA, great care should be taken when performing the posteromedial tibial bone cut. The power saw blade should be used with extreme caution and appropriate protection, rather than attempting one cut to remove the entire posterior tibia. Also, care is needed when performing TAA in patients with posttraumatic ankle arthritis because disease-induced anatomic alterations may increase vulnerability to injury.

If tibial nerve injury is suspected, surgical exploration should be done at the earliest opportunity to optimize prognosis. Prompt neurolysis is also needed to free the nerve from adhesions and to remove scar tissue inside and around the damaged nerve. Complete resection and reconstruction might be required if the nerve is beyond repair or after observation of a neuroma. However, despite prompt surgical treatment after complications, predicting the clinical outcomes according to the time of nerve repair is difficult, and close observation for symptoms is also necessary due to the high likelihood of ambiguous outcomes.

## 4 Conclusion

Tibial nerve injury is a rare complication of TAA, but can cause serious problems. Diagnosis may be delayed because these injuries are often unrecognized or misdiagnosed. If tibial nerve injury is suspected, surgical exploration should be performed without delay. Our results suggest that surgeons should take great care when cutting the tibia at the posteromedial corner of the ankle in patients with ankle osteoarthritis and deformities.

## Author contributions

**Conceptualization:** Keun-Bae Lee.

**Data curation:** Gun-Woo Lee.

**Investigation:** Gun-Woo Lee.

**Supervision:** Keun-Bae Lee.

**Writing – original draft:** Gun-Woo Lee.

**Writing – review & editing:** Keun-Bae Lee.
